# Intervention Implementation of Tools of the Mind for Preschool Children’s Executive Functioning

**DOI:** 10.3389/fpsyg.2021.624140

**Published:** 2021-03-11

**Authors:** Priscilla Goble, Toria Flynn, Cambrian Nauman, Pond Almendarez, Meagan Linstrom

**Affiliations:** ^1^School of Family and Consumer Sciences, Texas State University, San Marcos, TX, United States; ^2^Department of Educational Psychology, University of Texas at Austin, Austin, TX, United States; ^3^Dell Children’s Medical Center of Central Texas, Austin, TX, United States

**Keywords:** executive function, implementation, intervention, preschool, teacher characteristics, Tools of the Mind

## Abstract

One of the more prominent early childhood interventions focused on the development of executive function (EF) skills is Tools of the Mind (*Tools*; Bodrova and Leong, 2019). Intervention studies comparing *Tools* classrooms with control classrooms, however, reveal inconsistent findings for children’s EF outcomes. The current study utilizes Head Start CARES teachers assigned to the Tools of the Mind enhancement intervention *(Tools*; *N* = 75) and the children in their classrooms *(N* = 738). Relations between teachers’ characteristics (i.e., teaching experience, psychological well-being, and educational background), training attendance and implementation (i.e., coach rated fidelity and observed scaffolding), and the interaction among these factors were examined as predictors of classroom-level gains in EF. Results revealed several significant moderation effects indicating that *Tools* implementation is related to classroom EF gains for some but not all teachers.

## Introduction

Young children’s executive functioning (EF), or their ability to maintain focus, control impulses, and to think before acting, has been related to a host of positive adjustment outcomes (Ursache et al., 2012). Kindergarten teachers identify children’s EF abilities as essential to school readiness and success (Rimm-Kaufman et al., 2000). Thus, identifying school-based interventions that foster preschooler’s EF skills has been an area of considerable focus. One of the more prominent early childhood interventions focused on the development of EF skills is Tools of the Mind (*Tools*; Bodrova and Leong, 2019). Based on Vygotsky’s theory of development, *Tools* is a classroom curriculum enhancement designed to emphasize the role of teachers in facilitating children’s play (Bodrova and Leong, 2019). Through *Tools* training teachers learn how to help children develop a plan for play, as well as how to help them self-monitor, solve problems, and carry out their plan during play settings. Children’s planning of activities, retaining these plans in memory, and enacting them during pretend play are all actions intended to support key aspects of executive function (including mental flexibility, deliberate memory, focused attention, and inhibitory skills).

Intervention studies comparing Tools of the Mind (*Tools*) classrooms with control classrooms, however, reveal inconsistent findings for children’s EF outcomes. While some impact studies reveal no significant differences between the intervention and control groups on measures of EF, other studies have found that children in *Tools* classrooms do make significant gains in EF (see Baron et al., 2017). Researchers have hypothesized that inconsistencies in the impact of *Tools* could be due to variability in teacher characteristics and/or in the variability of implementation. The present study addresses these hypotheses utilizing a subset of data from Head Start CARES, a large randomized-control trial that implemented the Tools of the Mind enhancement (Morris et al., 2014). Specifically, we examined if variability in participating teachers’ characteristics (i.e., teaching experience, psychological well-being, and educational background) and intervention training(i.e., attendance) and implementation (i.e., coach rated fidelity and observed scaffolding) were related to children’s gains in EF skills.

## Tools of the Mind

Created by Russian psychologist Elena Bodrova and American psychologist Deborah Leong, Tools of the Mind (*Tools*) was developed to address a need for developmentally appropriate teaching techniques that foster foundational executive functions within a diverse population of children (Bodrova and Leong, 2001). Bodrova and Leong (2001) based *Tools* on Vygotsky’s theory that cultural tools enable the attainment of higher mental functions and are the most influential areas of learning and development (Bodrova and Leong, 2007). Vygotsky theorized that the outcome of social experiences is contingent on the mastering of these mental tools, in which children are able to use EF behaviors independently (Aras, 2015). Rather than providing explicit lessons on these skills, *Tools* changes the way that make-believe play and other learning experiences are structured and supported in the classroom.

Bodrova and Leong developed *Tools* over several years in four phases. The first phase was an initial attempt at developing teaching practices to fit the classroom environment using Vygotsky’s theoretical foundations (Bodrova and Leong, 2001). For example, Bodrova and Leong (2001) translated Vygotsky’s argument that make-believe play is contingent on children abiding by a set of rules in teacher practices that help children plan, remember, and enact rules during make-believe play. A major theme derived from Vygotsky’s theory, which underlies all *Tools* practices, is the role of scaffolding. Scaffolding is the gradual “release of responsibility” from the teacher to the learner and is described as the process of transition from assistance to independence (Wood et al., 1976). Accordingly, the *Tools* theory of change includes a three step process in which (1) teachers scaffold children’s EF development by providing children with cognitive “tools” (e.g., language), (2) the children regulate one another in shared activities using modeled “tools” (e.g., planning), and (3) these activities result in learning and skill development (e.g., EF; Farran and Wilson, 2014).

Phase two of development attempted to train a large number of teachers how to use the strategies developed in phase one and make adaptations (Bodrova and Leong, 2001). In phase three the developers experimented with methods of teacher training and directed the first quasi-experimental study that evaluated the effects of the intervention on student’s pre-literacy outcomes (Bodrova and Leong, 2001). Results from this phase revealed increased performance on measures of pre-literacy. In this initial study, Bodrova and Leong (2019) found that teachers with the strongest results were the ones who had higher degrees of fidelity; however, it is unclear how fidelity was measured. In the fourth phase, Bodrova and Leong (2019) continued the development of strategies and the application of strategies in diverse settings. The empirical evaluation completed during phase four found *Tools* to improve classroom quality and children’s EF (Barnett et al., 2008). The Barnett et al. (2008) evaluation was also the first randomized control trial to examine *Tools.*

Since the finalization of the Tools of the Mind, there have been several more studies examining the effects of *Tools* for children’s executive functioning. A meta-analysis of four randomized controlled trial (RCT) studies (Baron et al., 2017) and three more RCTs published after 2017 (Blair et al., 2018; Solomon et al., 2018; Diamond et al., 2019) examining *Tools* highlighted inconsistencies in the effectiveness of the intervention for children’s EF development. Although some RCTs have found that the Tools of the Mind intervention effectively promoted children’s executive function abilities (i.e., Diamond et al., 2007, 2019; Blair and Raver, 2014; Blair et al., 2018), others have not found effects of *Tools* on EF (i.e., Farran and Wilson, 2014; Morris et al., 2014). Researchers who conducted an RCT, in Canadian preschools, confirmed inconsistencies in the field and reported no main effect of *Tools* for EF skill development, but did find a significant moderation effect between *Tools* and children’s initial level of hyperactivity/inattention, indicating a benefit of *Tools* for some but not all children (Solomon et al., 2018).

The largest randomized-control trial examining *Tools*, the Head Start CARES study, evaluated the effects of three distinct interventions; these included Preschool PATHS, Incredible Years, and Tools of the Mind–Play, an adapted version of the *Tools* curriculum in which teachers were trained for only 1 year instead of 2 years, as is typical for *Tools*. The three interventions that Head Start CARES tested were selected because each was thought to exemplify a distinct theory of change for improving children’s social-emotional development. Specifically, Preschool PATHS focused on training teachers to use clearly outlined lessons and teaching strategies to improve children’s emotion knowledge and social problem-solving skills. Incredible Years focused teacher training on strengthening and promoting positive teacher-child relationships, classroom organization, and proactive discipline strategies. Tools of the Mind—Play focused on training teachers to scaffold children’s planning and enacting role-playing to strengthen children’s ability to regulate their emotions and behavior. While the interventions shared a core goal (improving children’s social-emotional competence), the CARES team hypothesized that each one had a different mediating or intervening pathway to social-emotional competence for children. The primary expected pathway for *Tools* was through the development of children’s executive function skills (Morris et al., 2014).

Contrary to study hypotheses, the Head Start CARES RCT showed that children in *Tools* classrooms did not demonstrate better EF skills than children in the control group (or children in the other two interventions). The authors speculated that this result was due to the difficulty of implementing *Tools*. Morris et al. (2014) note that the comprehensiveness of *Tools* and the amount of restructuring needed to implement it made *Tools* difficult to deliver with fidelity to the program as it was designed, even in its modified form for the Head Start CARES study. In this study, teachers assigned to the *Tools* condition scored the lowest on fidelity of implementation, compared to teachers assigned to the two other interventions. The authors hypothesize, as others have, that the inconsistent findings in the effectiveness of *Tools* for children’s EF was likely due to variation in teacher’s characteristics, intervention implementation, or a combination of these factors (Bodrova and Leong, 2019).

## Teacher Characteristics

Researchers have hypothesized that variability in teacher characteristics may impact the effectiveness of school-based interventions for improving children’s gains in EF skills (Downer et al., 2010). Indeed, teacher’s personal and professional characteristics have been linked both to children’s skill development (Bogard et al., 2008) and intervention implementation (Zaslow et al., 2010). The current study focuses on three key teacher characteristics: years of experience teaching, psychological well-being, and educational background.

Although findings are not robust to all areas of children’s development, there is evidence that teacher’s years of experience teaching is related to various child outcomes (McMullen et al., 2020). For example, Reilly and Downer (2019) found teacher years of experience to be positively related to observed gains in preschooler’s behavioral control across an academic year. Furthermore, intervention work has demonstrated that teaching experience is also related to intervention implementation. Illustratively, professional development intervention work with preschool teachers has demonstrated that teacher’s responsiveness to the intervention decreased as teachers reported higher levels of experience teaching (Downer et al., 2009). Their findings suggest that novice teachers are more receptive to intervention factors, and thus associated behavioral changes, than are more experienced teachers. As it applies to *Tools* implementation, teachers with less experience may be better able to integrate new ideas and practices within their classroom.

Another factor that likely impacts the effectiveness of an intervention for children’s EF skills is the teacher’s psychological well-being or levels of stress. A growing body of work indicates that the quality of interactions between preschool teachers and children suffers when teachers experience high levels of stress or burnout (Sandilos et al., 2018). Thus it stands to reason that if teachers are experiencing stress, they may not be as effective in positively impacting children’s EF skill development. Indeed, researchers have found a significant negative association between teacher stress and young children’s EF skill development from fall to spring (Neuenschwander et al., 2017). Conversely, providing teachers with additional skills and support through an intervention could buffer impacts of stress (Jennings and Greenberg, 2009). One study, using the Head Start CARES data, found that teachers who are professionally supported through interventions were less likely to demonstrate declines in instructional quality due to emotional burnout over the course of the year, compared to the control group (Sandilos et al., 2020). However, this buffering effect was only present for teachers trained in PATHS and Incredible Years interventions, not for those trained in *Tools*. Thus, it is unclear how teacher’s well-being and distress would impact the effectiveness of *Tools* for children’s EF skills.

The final teacher characteristic of focus is teacher’s educational background. Researchers consistently demonstrate that a bachelor’s degree in any subject or specialized training at the college level is related to competent teaching, intervention implementation, and positive child outcomes (Early et al., 2007). For example, a quasi-experimental trial in Head Start centers found that children whose teachers received a literacy-focused intervention made greater gains in phonological awareness when their teachers held a 4-years degree (Landry et al., 2006). Beyond having any 4-years degree, education professionals agree that teaching young children is a complex task, best performed when teachers have a detailed understanding of child development, curriculum, and pedagogy (Bowman, 2011). As evidence, studies have shown that teachers with formal education in Child Development (CD) demonstrated higher teaching quality and better child outcomes than teachers holding formal education in a different field (e.g., Burchinal et al., 2002). It is unknown, however, whether advanced training in CD would be more or less beneficial for teacher’s understanding of and implementation of *Tools* within their classrooms.

## Effective Implementation

When evaluating the success of programs and interventions, effective implementation is consistently associated with better outcomes (Durlak and DuPre, 2008). Only a few studies have measured *Tools* implementation and none have examined implementation as a potential factor contributing to children’s EF development (e.g., Diamond et al., 2007; Barnett et al., 2008; Solomon et al., 2018). A phenomenon that is not uncommon in school-based intervention work (Capin et al., 2018). In this study, we examined three aspects of implementation anticipated to affect the extent to which *Tools* produced intended effects on children’s EF development in the Head Start CARES study: training attendance, fidelity, and quality.

Training attendance, which refers to the attendance and participation of teachers in the training sessions leading up to the implementation of an intervention, plays an important role in effective intervention implementation (O’Donnell, 2008). For example, programs implemented in which teachers attended only a small portion of training sessions tend to be less successful than when the teachers attend all training sessions (Reyes et al., 2012). In fact, the value of the program or intervention being implemented may be threatened when numerous teachers are absent from the trainings, especially when key concepts or ideas are outlined (Dane and Schneider, 1998).

Typically measured through teacher report, fidelity, also called adherence, is the extent to which teachers deliver program components as prescribed (Greenberg et al., 2005). Research consistently suggests that intervention fidelity affects the program’s outcomes (Durlak and DuPre, 2008). For example, one study examining a school readiness intervention in Head Start classrooms, demonstrated a significant positive relation between intervention fidelity and children’s EF skill development (Marti et al., 2018). Fidelity is the only aspect of intervention implementation consistently measured in efficacy studies of *Tools* (e.g., Solomon et al., 2018). Consistent with previous research, the Head Start CARES study measured fidelity as how well teachers implemented activities, strategies, and other programmatic activities specific to *Tools*. Bodrova and Leong (2019) have reported variability in teacher’s adherence (or fidelity) to *Tools*. Although, fidelity has never been examined as a predictor of children’s EF development, it is often hypothesized that variation in the degree to which teachers adhere to the components of *Tools* may have led to the inconsistent results across previous studies. In other words, the more accurately a teacher is able to replicate of the original *Tools* model, the more implementation of *Tools* is likely to impact children’s EF development.

Another defining factor of effective implementation, quality, refers to the means by which a program is being executed (Dane and Schneider, 1998). Although training attendance and fidelity are widely accepted with consistent conceptualization and measurement across studies and fields, quality of program delivery is more program specific (Hamre et al., 2010). Intervention implementation work by Hamreet al. (2010) indicates that program specific measures of implementation quality have been associated with greater gains in preschooler’s skill development. A primary goal of *Tools* is to train teachers to provide individualized scaffolding to children throughout the various stages of play (i.e., planning, retaining plans in memory, and enacting plans during play; Bodrova and Leong, 2007). Accordingly, in this study quality assessed independent observer’s ratings of the lead teachers’ scaffolding practices.

## Present Study

The aim of the present study was to address important gaps in our understanding of how natural variability, and variability due to teacher characteristics, in *Tools* training and implementation impacts the effectiveness of *Tools* for children’s EF development. Specifically, teacher’s training attendance and implementation (i.e., fidelity and quality) of *Tools* were examined as predictors of variability in classroom-level gains in EF skills across a preschool year. In addition to training attendance and effective implementation, researchers have hypothesized that variability in teacher characteristics may influence the effectiveness of *Tools* for children’s EF (Morris et al., 2014). We identified three teacher characteristics in the current study as potential moderators of the relations between *Tools* implementation and EF outcomes: (1) years of experience, (2) psychological well-being, and (3) a degree in child development (CD) or a related field. We address two research questions: (1) to what extent are teacher characteristics, training attendance, fidelity, and quality of *Tools* implementation associated with classroom-level growth in EF skills across the preschool year? and (2) to what extent are relations between implementation and classroom outcomes moderated by teacher characteristics?

## Materials and Methods

We use data from Head Start CARES, a large randomized-control study, which evaluated the effects of three distinct classroom-based interventions. The evaluation was conducted by randomly assigning approximately 100 Head Start centers to one of four groups that received one of three interventions or served as the control condition. The current study examined only teachers who received the Tools of the Mind—Play classroom intervention. The Head Start CARES study condensed *Tools*, normally a 2 years program, into a 1 year enhancement.

### Participants

Participants were preschool teachers recruited from 17 Head Start grantees across 10 states, selected to reflect the geographic, racial, and ethnic diversity of the national Head Start population. Each grantee had between four and 12 Head Start centers and each center had between one and six participating classrooms. A total of 104 centers across 22 blocks were randomly assigned to one of the four conditions^1^. In the end, 75 teachers/classrooms and 7,384 years old children were assigned to the *Tools* intervention.

Of the 75 teachers, 100% were female (*M* age = 43 years), the majority had at least a bachelor’s degree (57%) and had been teaching for 10 years or more (67%). Approximately half of the children in teachers’ classrooms were girls (51%) and a little over half of the classrooms were full day programs (68%). Teachers were Black non-Hispanic (28%), White non-Hispanic (30%), and Hispanic (32%) with 10% of teachers identifying as multi-racial, Asian, Native American, or Other non-Hispanic. On average classrooms were characterized by 4-years-old (*M* = 54 months, range 45–61 months) mostly Black (39%) and White (41%) children with 31% identifying as Latinx. There were approximately 18 children in each classroom (range 9–27) with an average teacher-child ratio of 1:9. Of these children, on average 13 children (range 3–21) per classroom participated in the study.

### Procedures

Data utilized in the current study were collected using multiple methods at multiple time points, including the spring before the preschool year in which *Tools* was implemented and throughout the preschool implementation year. In the spring of the year prior to implementation, teachers reported on their demographic characteristics and well-being. Throughout the year, *Tools* coaches and independent observers reported on *Tools* implementation factors (i.e., training attendance, fidelity, and quality/scaffolding). Direct assessments of children’s EF skills occurred in the fall and spring of the implementation year.

To promote effective implementation and support teachers, a *Tools* coach (*N* = 17) was assigned to each classroom. Lead teachers of the classrooms had the opportunity to attend five to six *Tools* trainings the summer prior to and throughout the implementation year. Coaches collected attendance forms during teacher trainings. The coach was responsible for attending their assigned classrooms’ training sessions with the teachers. In addition, the coaches were required to spend 90 min in each classroom every week- this included 30 min with the teacher and 60 min observing and rating fidelity. Independent observers, separate from coaches, observed each classroom for a 2 h period. These observers did not know about the intervention and were asked to rate the lead teachers’ scaffolding practices using a five-point Likert scale. Independent assessors, trained to assess children’s executive function skills, directly assessed children in the fall and spring of the preschool year.

### Measures

#### Teacher Characteristics

Information about teacher demographic and background characteristics (i.e., teacher’s years of experience, psychological well-being, and educational background) was collected from the Teacher Self-Survey. Teachers completed the Teacher Self-Survey in the spring of the year prior to implementation.

##### Years of Experience Teaching

Teachers reported on the total amount, in years and months, of experience they have teaching. If the sum of the years and months reported by the teachers was greater than 10 years of experience, then their years or teaching experience was coded as a positive response (i.e., 0 = <10 years; 1 = >10 years). This coding decision was made to protect the identity of the participants and the binary variable is the only available data point for years of experience in the available data.

##### Psychological Distress

Teachers also reported on their psychological well-being using the Kessler Psychological Distress Scale K-6 (Kessler et al., 2003). The K-6 is a shortened well-validated 6-item version of the K10 and is preferred when screening for mood or anxiety disorder due to its brevity and consistency across sub-samples (Kessler et al., 2002). The K-6 contains items such as, “during the last 30 days, about how often did you feel hopeless?” and “during the last 30 days, about how often did you feel so depressed that nothing could cheer you up”. Response choices were based on a 5- point Likert-Type scale from “1 (none of the time)” to “5 (all of the time)” and these were rescaled to a scale of “0 (agree strongly)” to “4 (disagree strongly).” We created as total sum score that ranged from 0 to 24. Low scores indicated low levels of teacher psychological distress and high scores indicated high levels of teacher psychological distress. K6 has been found to be reliable with Cronbach’s α ranging from 0.89 to 0.92 (Kessler et al., 2003) and has little bias concerning education and sex (Baillie, 2005).

##### Educational Background

Teachers responded yes or no to the following statement “teacher obtained their highest degree in Child Development/Developmental Psychology, Early Childhood Education or a related field.”

#### Tools Intervention Training and Implementation

##### Training Attendance

Coaches recorded teachers’ attendance at Tools of the Mind workshops. *Tools* workshops occurred over 2–3 days in the summer before the start of the school year, 1 day during the school day sometime in October, 1 day = during the school year in January, and 1 day during the school year in April/May. Training attendance was a percentage calculated as the number of training days attended by the lead teacher out of total number of training offered.

##### Fidelity

A trained local coach met with the teachers every week in order to observe the teachers in the classrooms (Mattera et al., 2013). Coach Monthly Fidelity Logs (MIS): At the end of each month, coaches reported extensively about their teachers and classrooms, including the teachers’ response to enhancement-specific coaching, consultation, and implementation; modeling and generalization of the enhancement throughout the school day; fidelity of teaching and supporting children; fidelity of programmatic activities; organizational support; and the co-teacher relationship. A single score referring to fidelity to the enhancement as delivered in the classroom was calculated each month. The current study uses a mean composite of all monthly fidelity scores, representing the coach rating of fidelity across the full year.

##### Quality

Lead teachers’ practices were measured with the Adapted Teaching Style Rating Scale (Adapted TSRS; Mattera et al., 2013). In the spring before implementation (baseline) and in the spring of the implementation year (follow-up), observers who were not informed of the intervention status of the classrooms observed the lead teacher in each classroom for a 2 h observation period. Teachers were rated on three subscales: (1) classroom management, (2) social-emotional instruction, and (3) scaffolding (a central component of the Tools of the Mind enhancement). The *scaffolding* subscale assesses teachers’ use of scaffolding— a practice that supports a child’s activity or response at his or her current level of understanding while extending the activity or response in order to help the child advance to the next level of ability. In this case, the teacher’s practice was coded for instances of scaffolding of (1) children’s pretend play by supporting their planning of that activity and expanding the play as it is being enacted, and (2) interactions between children when they are playing together. For the current analyses, a change score was calculated to reflect the gains teacher’s made in scaffolding due to participation in the intervention. Specifically, scaffolding at baseline was subtracted from scaffolding at follow-up.

#### Children’s Executive Function

##### Head-to-Toes

Head-to-Toes, a simplified version of Head-Toes-Knees-Shoulders (HTKS) and a commonly used measure of children’s EF, is a task in which children play a large motor game involving paired verbal rules (Ponitz et al., 2008): “touch your head” and “touch your toes.” Children first responded to the verbal commands as spoken and then were instructed to switch and respond in an “opposite” way (such as touching their head when told to touch their toes). The activity is intended to tap children’s ability to suppress a dominant response (to follow the assessor’s directions) in order to carry out a subdominant response (to do the opposite of what the assessor asks them to do) and draws on three executive functions: inhibitory control, attention skills, and working memory. The Head-to-Toes task includes 10 trials, and assessors score each trial as “correct,” “incorrect,” or “self-correct” if the child starts to perform the incorrect action but then catches himself or herself and ultimately performs the correct action. Children are scored on the number of trials they answer correctly out of the 10 trials. In Head Start CARES, “self-correct” responses were recoded as “correct,” and each item was scored as a 0 or 1. The range of the measure is 0–10, with a 0 indicating that the child got no trials correct and a 10 indicating that the child got all 10 trials correct. The Head-to-Toes task has demonstrated validity and inter-rater reliability in early childhood (Ponitz et al., 2008). Scores on the Head-to-Toes task significantly correlate with teacher report of behavior regulation items on the Child Behavior Rating Scale (*r* = 0.15–0.47; Ponitz et al., 2008).

##### Pencil Tap

Pencil Tap, a direct assessment of inhibitory control, was an adapted version of a standard peg-tapping task, which used pencils rather than pegs (adapted from Luria, 1966; Diamond and Taylor, 1996; see Smith-Donald et al., 2007). This task requires children to inhibit a natural tendency to mimic the action of the experimenter while remembering the rule for the correct response, and is thought to assess inhibitory control, attention skills, and working memory. The Pencil Tap task also requires greater fine-motor skills than the Head-to-Toes task, mentioned above. In this task, an independent assessor asks the child to tap on a table twice with a pencil when the assessor taps once, and once when the assessor taps twice (Diamond and Taylor, 1996). This task begins with a series of practice and coaching trials to ensure that the child understands the rules of the “game” before the assessment begins. The assessor’s final judgment of whether the child understands the rules was recorded in a checkpoint. The assessor is instructed to skip the assessment if the child does not understand the rules of the game. Children are scored on the proportion of trials they answer correctly out of 16 trials. Assessors scored children’s response to each trial on a scale of 0–3 (where 0 = child doesn’t tap, 1 = child taps once, 2 = child taps twice, 3 = child taps more than two times). Each trial was then recoded to 1 if the child answered correctly and coded as a 0 if the child answered incorrectly. The final score is the proportion of trials the child gets correct. If the child did not understand the rules of the game and skipped the assessment, the final score was coded as a 0. Scores were calculated if 25% or fewer of the items were missing. Children got an average of 46% of responses correct at baseline. Nationally, Head Start children get an average of 43% of responses correct (Moiduddin et al., 2012). Percent of correct responses on this assessment has demonstrated good concurrent and construct validity with other measures of inhibitory control as well as predictive validity for school readiness outcomes such as phonemic awareness (Blair and Razza, 2007; Smith-Donald et al., 2007).

#### Covariates

We included four covariates, the Head Start centers, whether the Head Start classroom was full day, the percent of boys in the classroom, and the teacher-child ratio, because they are conceptually related to EF skills. Additionally, to examine change in children’s scores on measures of EF using residualized change scores, we entered the time 1 (fall) EF measure as a predictor and used the corresponding time 2 (spring) EF measure as the outcome in each model.

### Data Analysis

We used M*plus*7 (Muthén and Muthén, 1998–2012) to examine the research questions. Of the 738 children, 78 children (10.6%) had missing data on executive function outcomes measured in the spring. Head Start CARES only provides children’s demographic characteristics aggregated to the classroom level to protect the identity of individual children; thus, prohibiting missing data analyses comparing children with complete data to those with missing data on EF outcomes. At the aggregate classroom level there was between 0 and 50% missing (*M* = 11% missing)EF data. To address missing data, all models were estimated using a Full Information Maximum Likelihood (FIML) estimator for the full sample (*N* = 738).

Children (Level1) were nested within teachers (Level 2) and teachers were nested within intervention coaches (Level 3). Given our interest in examining teacher/classroom-level predictors, intraclass correlations (ICCs) of the child-level EF outcome variables were examined at Level 2. The ICCS were 0.06–0.00 for Head-to-Toes and Pencil Tap, respectively. Despite the low ICCs, the clustered data structure should not be ignored, especially when the unit of interest is the level-2 unit (e.g., teacher/classroom; Huang, 2018). Thus, we specified a means-as-outcomes model utilizing Mplus command “type = three level,” which provides scaled standard errors robust to non-independence and non-normality. Level 1 predictors included children’s scores on measures of EF in the fall and were estimated as fixed effects. Head Start center, intervention coach, whether the classroom was full day, teacher-child ratio, and classroom average for percentage of boys, along with the study predictors reflecting teacher characteristics (i.e., years of experience, distress, and CD degree) and implementation (i.e., training attendance, fidelity, quality/scaffolding) were analyzed at Level 2. No variables were examined at Level 3.

Utilizing a traditional regression framework, we took a stepwise approach to examining the research questions. First, we estimated a model only including the covariate variables and teacher characteristics main effects (i.e., teacher’s years of experience, distress, and whether or not they held a CD related degree). Next, to explore the associations between *Tools* implementation on residualized change in children’s EF outcomes, we specified a model with all covariates, teacher characteristics, and implementation main effects (i.e., training attendance, fidelity/adherence, quality/scaffolding). Finally, to explore the moderation effects of teachers’ characteristics (i.e., experience, distress, CD degree) on the relations between *Tools* implementation and residualized change in classroom level EF outcomes, interaction effects were tested in a third model including the main effects and covariates (i.e., 3 implementation factors by 3 teacher characteristics = 9 interaction effects). All continuous predictors were grand-mean centered (Enders and Tofighi, 2007). To determine significance of the simple slopes for significant moderated effects we used the online interactive calculator for probing multilevel interactions developed by Preacher et al. (2006).

## Results

Preliminary analyses conducted using SPSS Version 24 examined the descriptive statistics, skewness, and kurtosis of all study variables for the full sample study of teachers (*N* = 75) assigned to the Tools of the Mind enhancement intervention and the children in their classrooms (*N* = 738; see Table 1). Overall, the data were normally distributed. Correlations between and among EF measures at pre- and post-intervention revealed stable estimates (see Table 2). Notably, there were several significant correlations among the study predictors. As can be seen in Table 3, teacher’s years of experience was positively related to training attendance and implementation fidelity and negatively related to implementation quality (i.e., scaffolding). There were also significant positive relations between teacher’s psychological distress and implementation fidelity and quality. There were few significant relations between the study predictors and EF outcomes measured post-intervention (see Table 3). A significant positive relation between a CD degree and Head-to-Toes suggests that teachers with a degree in CD or related fields had a slightly higher classroom average on EF at the end of the year.

**TABLE 1:** Descriptive statistics for classrooms and children.

	N	%	*M*	*SD*	Min	Max	Skew	Kurtosis
**Covariates**								
Full day	67	68.7						
% Boys	65		0.53	0.14	0.26	1.18	1.44	5.33
Teacher/child ratio	65		9.14	2.66	4.25	19.00	1.56	4.27
Head-to-toes (pre-intervention)	475		2.40	3.59	0.00	10.00	1.27	–0.06
Pencil tap (pre-intervention)	476		0.46	0.35	0.00	1.00	0.05	–1.42
**Teacher characteristics**								
Experience	68	66.2						
Distress	68		3.02	3.54	3.02	21.00	2.51	9.78
Child development (CD) degree	60	73.3						
**Training and implementation**								
Attendance	75		0.82	0.23	0.20	1.00	–1.06	0.11
Fidelity	75		3.25	0.89	1.33	4.88	–0.49	–0.58
Scaffolding	73		0.24	0.87	–2.50	2.75	–0.02	1.51
**Outcomes**								
Head-to-toes (post-intervention)	660		4.12	4.27	0.00	10.00	0.35	–1.67
Pencil tap (post-intervention)	660		0.68	0.31	0.00	1.00	–0.76	–0.71

*Experience = teacher’s years of experience teaching (0 = <10 Years; 1 = >10 years). CD Degree = Child Development Degree. Valid N (listwise) for Outcomes = 657.*

**TABLE 2:** Correlations between and among children’s pre-intervention and post intervention executive function outcomes.

		1	2	3
**Pre-intervention (fall)**				
1	Head-to-toes	−		
2	Pencil tap	0.46***	−	
**Post-intervention (spring)**				
3	Head-to-toes	0.52***	0.47***	–
4	Pencil tap	0.28***	0.51***	0.38***

Degrees of freedom ranged from 429 to 660. ***p ≤ 0.001.

**TABLE 3:** Correlations between and among teacher characteristics, tools implementation, and children’s executive function outcomes.

		1	2	3	4	5	6
**Teacher characteristics (pre-intervention)**							
1	Experience	−					
2	Distress	−0.22***	−				
3	Child development (CD) degree	0.03	–0.01				
**Training and implementation**							
4	Attendance	0.13***	–0.01	–0.07	−		
5	Fidelity	0.11**	0.17***	0.03	0.28***	−	
6	Scaffolding	−0.15**	0.11**	–0.03	0.30	0.08*	−
**Post-intervention outcomes**							
7	Head-to-Toes	–0.04	–0.02	0.09*	–0.02	0.03	–0.03
8	Pencil Tap	–0.04	0.05	0.04	0.00	0.01	0.00

Experience = teacher’s years of experience teaching (0 = <10 Years; 1 = >10 years). Degrees of freedom ranged from 529 to 1,003. *p < 0.05; **p < 0.01; ***p ≤ 0.001.

Based on previous implementation work (Bodrova and Leong, 2019), we expected that the main effects of training attendance, fidelity, and quality (i.e., scaffolding) would be positively related to classroom gains in children’s EF skills. Consistent with our hypotheses, main effects models including the covariates revealed a significant positive relation between coach rated fidelity to *Tools* and classroom gains on the Head-to-Toes measure of EF (β = 0.54, *p* < 0.05). This effect was not found for Pencil Tap. Additionally, and contrary to our expectations, there were no significant main effects of *Tools* training attendance or implementation quality (i.e., quality) on classroom gains in EF. There was also a significant main effect of teaching experience on classroom gains in EF (i.e., Head-to-Toes only; β = −0.36, *p* < 0.05).

In the models examining our second research question, we found significant moderation for several implementation by teacher characteristic interactions on EF development measured with the Pencil Tap but not Head-to-Toes (see Table 4). Teacher’s years of experience teaching moderated the relation between training attendance and classroom gains in EF. Teacher’s years of experience also significantly moderated the relation between implementation fidelity and classroom-level EF gains. Additionally, teacher’s educational background (i.e., CD degree or other) moderated the relation between implementation quality (i.e., scaffolding) and classroom-level EF gains.

**TABLE 4:** Fixed effects estimates (top) and variance estimates (bottom) for final means-as-outcomes multilevel regression model on gains in executive function.

	Head-to-toes		Pencil tap	
Intercept	0.92	(0.69)	4.88	(1.64)**
**Level-1 Fixed effects**				
Outcome (pre-intervention)	0.52	(0.04)***	0.53	(0.03)***
**Level-2 Fixed effects**				
Full day	–0.20	(0.16)	–0.27	(0.20)
% Boys	0.09	(0.09)	–0.12	(0.16)
Teacher/child ratio	–0.07	(0.10)	0.04	(0.15)
Experience	–0.18	(0.09)	–0.17	(0.20)
Distress	–0.12	(0.10)	0.00	(0.11)
Child development (CD) degree	0.15	(0.09)	0.09	(0.15)
Attendance	–0.10	(0.14)	0.41	(0.174)*
Fidelity	0.44	(0.22)*	0.23	(0.26)
Scaffolding	–0.61	(0.11)***	–0.16	(0.27)
Attendance × experience	0.09	(0.16)	–0.39	(0.13)**
Attendance × distress	–0.05	(0.11)	0.12	(0.16)
Attendance × CD degree	0.07	(0.11)	–0.09	(0.15)
Fidelity × experience	–0.07	(0.11)	–0.42	(0.21)*
Fidelity × distress	0.05	(0.09)	0.11	(0.18)
Fidelity × CD degree	–0.22	(0.17)	0.10	(0.167)
Scaffolding × experience	0.29	(0.17)	0.05	(0.36)
Scaffolding × distress	–0.14	(0.12)	–0.18	(0.13)
Scaffolding × CD degree	0.24	(0.16)	0.39	(0.18)*
**Random effects**				
Level-1 residual	0.73	(0.04)***	0.72	(0.03)***
Level-2 intercept	0.01	(0.26)	0.01	(0.64)
Level-3 intercept	5.45	(2.48)	101.60	(4286.14)
**Pseudo *R*^2^**				
Level-1	0.27	(0.04)***	0.28	(0.03)***
Level-2	0.99	(0.26)***	0.99	(0.64)

Outcome (pre-intervention) = corresponding outcome (Head-to-Toes, Pencil Tap) assessed pre-intervention. Experience = teacher’s years of experience teaching (0 = <10 Years; 1 = >10 years). CD Degree = Child Development Degree. Center was controlled as fixed effects. Standardized beta estimates (standard errors) and p-values are reported. *p < 0.05; **p < 0.01; ***p ≤ 0.001.

**FIGURE 1:**
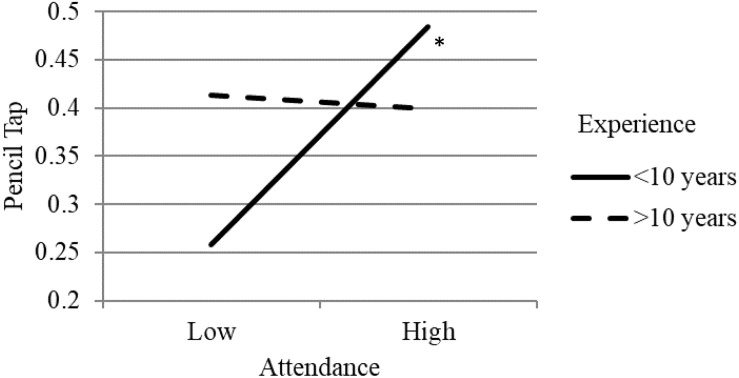
Moderated effect of training attendance on children’s pencil tap scores by teacher years of experience. ^∗^*p* < 0.05.

**FIGURE 2:**
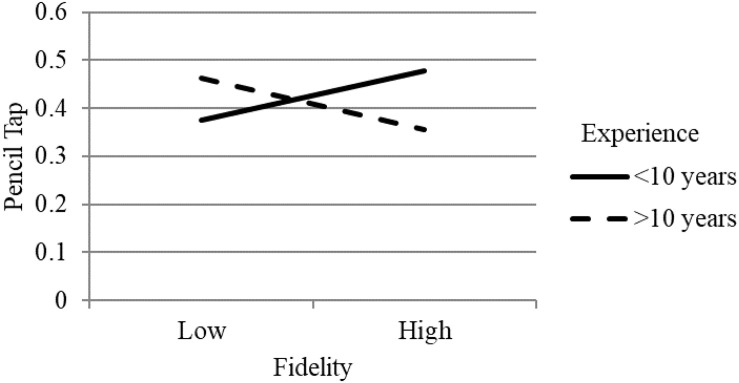
Moderated effect of implementation fidelity on children’s pencil tap scores by teacher years of experience.

**FIGURE 3:**
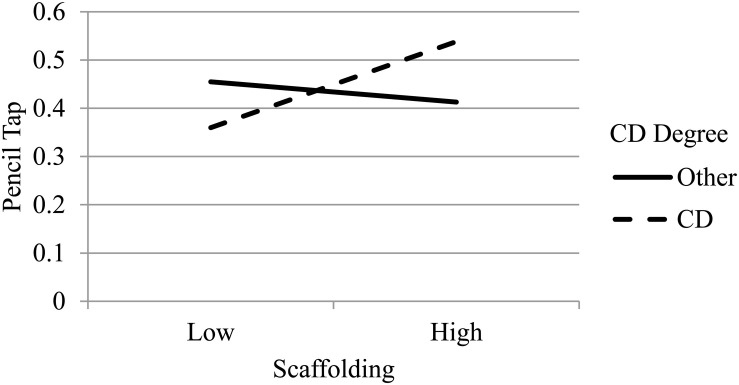
Moderated effect of implementation quality (scaffolding) on children’s pencil tap scores by teacher educational background. CD, Child Development.

Follow-up analyses examining the simple slopes for moderated effects showed that the relation between training attendance and classroom-level EF gains was significantly positive for teachers with less than 10 years of teaching experience (β = 0.28, *p* < 0.05) and was not related for teachers who had more than 10 years of experience (β = −0.02, *p* > 0.05; see Figure 1). The pattern of effects were similar for the interaction between implementation fidelity and teaching experience, indicating a positive relation between fidelity and EF gains for teachers with less experience; however, neither simple slope was statistically significant (see Figure 2)^2^. For the interaction between implementation quality (i.e., scaffolding) and educational background (i.e., CD degree or other), results suggested that there was a positive relation between quality and EF gains for teachers with a CD degree and not for teachers with a different degree, however, neither of the simple slopes were significant (see Figure 3). Non-significant simple slopes suggest that while the relations between implementation and classroom-level EF gains significantly differed due to teacher experience and educational background, the relations did not reach statistical significance for any group.

## Discussion

The current study examined relations between training attendance implementation (i.e., fidelity and quality) of *Tools* and executive function gains from fall to spring in preschool classrooms. Consistent with hypotheses, our study found that fidelity to *Tools* was related to classroom-level gains in EF. Additionally, several significant moderation effects emerged. Overall, the main effect and moderation results highlight the importance of considering intervention implementation and recipient characteristics (i.e., teachers) when examining the effects of the Tools of the Mind intervention for student outcomes.

Teacher’s years of experience teaching and their general well-being, assessed in the spring prior to intervention implementation, were significantly associated to teacher’s training attendance, implementation fidelity, and implementation quality (i.e., scaffolding). The results indicated that teachers with more experience (i.e., > 10 years) had higher attendance at *Tools* training and higher adherence to *Tools*, compared to novice teachers with less experience (i.e., <10 years). These findings differ from professional development (PD) intervention work demonstrating that teacher’s responsiveness to interventions (i.e., time spent engaging with online PD materials) decreased as teachers reported higher levels of experience teaching (Downer et al., 2009). Yet other researchers have shown that Head Start teachers’ intervention implementation (i.e., utilization and integration of materials and strategies) was not affected by the amount teaching experience they had (Wasik et al., 2006). These contradictions may be due to differences in how teaching experience is measured across studies or the content and complexity of the interventions. Nevertheless, the inconsistent findings highlight a need for more research that examines teacher’s years of experience teaching for intervention training and implementation.

With regard to quality, correlations revealed that novice teachers were more likely than teachers with more experience to increase their scaffolding of children’s play. This effect may be partially due to the fact that experienced teachers were already doing more scaffolding than novice teachers at baseline (*M* = 1.69 and 1.44, respectively). Nevertheless, the finding supports previous research by Downer et al. (2009) showing that novice teachers change their practices more than experienced teachers. There were also significant associations between teacher’s well-being and implementation fidelity and quality. Interestingly, it seems that higher levels of psychological distress in the spring prior to intervention implementation was related to higher levels of adherence to *Tools* and improved scaffolding. The prosocial classroom model (Jennings and Greenberg, 2009) suggests that teachers who are stressed are likely to experience burnout and not be able to meet the challenges of teaching. According to this framework, we might expect teachers with higher levels of distress to be less successful at implementing an intervention; however, recent research suggests that intensive teacher training and professional development can actually attenuate the negative association between poor teacher well-being and preschool teacher’s instruction (Sandilos et al., 2018).

In analyses addressing the first research question regarding the extent to which teacher characteristics, training attendance, fidelity, and quality of *Tools* implementation were associated with classroom-level growth in EF skills across the preschool year, results were inconsistent. There was a significant main effect for teaching experience on EF gains, suggesting that Tools classroom’s with more novice teachers (i.e., less than 10 years of experience) made greater gains in EF than classrooms with more experienced teachers. This finding contradicts previous research that teachers with more experience had classrooms with higher regulatory abilities (Reilly and Downer, 2019). However, the prior research did not explore this relation within an intervention as was done within the current study. There were no main effects for the other teacher characteristics (i.e., well-being and educational background).

Consistent with our hypotheses, results revealed a significant main effect between implementation fidelity and EF gains. These findings are consistent with other intervention work in Head Start classrooms that demonstrated a significant positive relation between intervention fidelity and children’s EF skill development (Marti et al., 2018). Importantly, this is the first study to statistically demonstrate the often hypothesized relation between *Tools* fidelity and EF gains (Bodrova and Leong, 2019). Contrary to our hypotheses and previous research, there were no main effects for training attendance or implementation quality. Although the current study did not support a direct relation between Tools training attendance and classroom EF gains, attendance was significantly related to EF gains for some teachers, discussed below. Similarly, teacher’s scaffolding was not directly related to EF gains.

In analyses examining our second research question regarding the extent to which relations between implementation and classroom outcomes were moderated by teacher characteristics, several significant relations emerged. First, there was a significant positive relation between training attendance and classroom EF gains for novice teachers but not for more experienced teachers. Although not statistically significant, there was also a positive association between implementation fidelity and EF gains for novice teachers. Together these findings suggest that more causal research should examine teaching experience as a factor in the impact of *Tools* for improving children’s EF. It is possible that because teachers with less experience have fewer resources than more experienced teachers, they are more open to interventions that provide new strategies. Novice teachers may also be less likely than tenured teachers to have long standing routines that would require unlearning. Indeed, previous research has shown that teachers with more experience are less likely to implement interventions (Witt et al., 1984; Ghaith and Yaghi, 1997) and find interventions less acceptable (Witt and Robbins, 1985).

Interestingly, the pattern of relations between implementation and EF gains was negative for more experienced teachers, although not statistically significant. For teachers with more than 10 years of experience teaching, higher levels of adherence to *Tools* was related to fewer gains for their students’ EF skills. We can only speculate about these counterintuitive findings. One possibility is that tenured teachers feel overwhelmed by the intervention because it differs from their established routines. Even for those teachers who implemented *Tools* with a high degree of fidelity and quality, the classroom-level EF skills ultimately suffered due to overwhelmed and stressed teachers. This finding may be uniquely related to the high level of difficulty implementing *Tools* (Morris et al., 2014). One study examining three teacher-focused interventions for teachers, including *Tools*, researchers found that while other interventions reduced the negative effect of teacher reported stress on teaching quality, *Tools* did not (Morris et al., 2014).

Teacher’s educational background significantly moderated the relation between implementation quality (i.e., scaffolding) and classroom gains in children’s EF skills. The findings revealed a positive, albeit non-significant, relation between teacher’s implementation quality/scaffolding and EF skill development for teachers who held a degree in CD or a related field. As previously noted, teachers’ education level and training has been linked to teacher interactional quality (Downer et al., 2010). The current findings suggest that when teachers are formally trained in CD, their pupils may benefit more from teacher-focused interventions. It is possible that *Tools* implementation had a stronger impact for teachers with CD training because they began the training with a stronger understanding about children’s development.

The main effect results for years of teaching experience and fidelity to *Tools* suggest that the overall intervention effects in RCTs, such as the one conducted by Head Start CARES, may benefit from reanalysis considering teacher and implementation characteristics as moderators (Morris et al., 2014). Furthermore, significant moderation effects suggest statistical differences in the relations between implementation and EF gains at various levels of teacher characteristics. Nevertheless, many of the simple slope analyses did not reach statistical significance and should be interpreted with caution. To the end, given the correlational nature of the analyses, all results should be interpreted with caution as they are they are exploratory and hypothesis-generating. Surprisingly, teacher’s psychological distress was not directly related to classroom-level EF nor did it moderate the relation between training and implementation of *Tools* and EF gains. As noted, teacher’s well-being was measured in the spring of the year preceding *Tools* training and implementation. The effect of teacher’s well-being on children’s outcomes may be better assessed simultaneously in future studies.

There are other measurement limitations worth noting related to the use of the secondary Head Start CARES data. For example, previous studies examining teacher interventions measure teachers’ years of experience differently (e.g., 0–3 or 0–5 years as unexperienced) compared to how the current study measured teachers’ years of experience (Domitrovich et al., 2009). Although there is not one operationalized definition of an experienced teacher we were limited by the availability of only a dichotomous years of experience variable. To this point, the current study utilized teacher’s training attendance as a measure of implementation in examining classroom-level EF gains. The measure used in the current study is consistent with studies of implementation dosage evaluating professional development interventions on change in teacher practices (Pianta et al., 2014). However, studies examining implementation dosage on child outcomes typically include measures of children’s exposure to the intervention (Domitrovich and Greenberg, 2000). A measure that assessed dosage of *Tools* strategies children actually received would have been a more direct measure of implementation. Similarly, observed change in scaffolding was the only measure of quality although scaffolding is only one of several strategies that *Tools* teachers were expected to implement. Future implementation research would benefit from analyses that utilize years of teaching experiences as a continuous variable, dosage children received rather than training teachers received, and measures of quality for all *Tools* strategies (e.g., planning).

Despite these limitations, this study had several strengths. First, where previous studies measuring *Tools* fidelity and quality have relied on teacher reports, trained local coaches and objective observers rated teacher attendance, fidelity, and scaffolding in the current study. Mackay (2013) reported that teachers’ perceptions and variation in the degree to which they are adhering to the components of *Tools* likely impacts their teacher self-reports, leading to biased measures of implementation. Observational assessments by trained coaches and objective observers provide unbiased insight into the fidelity and quality of program implementation.

The current study extends previous *Tools* research by examining teacher characteristics as moderators of the relations between implementation and classroom-level outcomes. Results revealed that the degree to which teachers implemented *Tools* was related to children’s EF development, especially for some teachers. This demonstrates that inconsistencies in the impact of the *Tools* could be due to both implementation and teacher characteristics.

## Data Availability Statement

The data analyzed in this study is subject to the following licenses/restrictions: Head Start CARES data—requires application for access through ICPSR. Requests to access these datasets should be directed to https://www.icpsr.umich.edu.

## Ethics Statement

Texas State IRB approved secondary data analysis for the current study. Texas State IRB is not the approving group for the orginal research. The original research was conducted by Office of the Administration for Children & Families. Written informed consent to participate in this study was provided by the participants’ legal guardian/next of kin.

## Author Contributions

All authors listed have made a substantial, direct and intellectual contribution to the work, and approved it for publication.

## Conflict of Interest

The authors declare that the research was conducted in the absence of any commercial or financial relationships that could be construed as a potential conflict of interest.

## Footnotes

1 In centers with five or six classrooms, all classrooms participated in the study. In centers with more than six classrooms, researchers randomly selected five classrooms.

2 Although not hypothesized, given the relations between teacher characteristics and fidelity and between fidelity and EF, we tested several indirect models in which teacher characteristics related to EF gains via *Tools* fidelity. None of the indirect effects were significant and thus were not included.
